# Integrated (Meta) Genomic and Synthetic Biology Approaches to Develop New Biocatalysts

**DOI:** 10.3390/md14030062

**Published:** 2016-03-21

**Authors:** María L. Parages, José A. Gutiérrez-Barranquero, F. Jerry Reen, Alan D.W. Dobson, Fergal O’Gara

**Affiliations:** 1 BIOMERIT Research Centre, School of Microbiology, University College Cork, National University of Ireland, Cork, Ireland; maria.lopezparages@ucc.ie (M.L.P.); jgutierrez@ucc.ie (J.A.G.-B.); j.reen@ucc.ie (F.J.R.); 2 School of Microbiology, University College Cork, Cork, Ireland; a.dobson@ucc.ie; 3 School of Biomedical Sciences, Curtin Health Innovation Research Institute, Curtin University, Perth WA 6845, Australia

**Keywords:** synthetic biology, metagenomics, biocatalysis, marine, biodiscovery, chassis, vector, heterologous expression

## Abstract

In recent years, the marine environment has been the subject of increasing attention from biotechnological and pharmaceutical industries as a valuable and promising source of novel bioactive compounds. Marine biodiscovery programmes have begun to reveal the extent of novel compounds encoded within the enormous bacterial richness and diversity of the marine ecosystem. A combination of unique physicochemical properties and spatial niche-specific substrates, in wide-ranging and extreme habitats, underscores the potential of the marine environment to deliver on functionally novel biocatalytic activities. With the growing need for green alternatives to industrial processes, and the unique transformations which nature is capable of performing, marine biocatalysts have the potential to markedly improve current industrial pipelines. Furthermore, biocatalysts are known to possess chiral selectivity and specificity, a key focus of pharmaceutical drug design. In this review, we discuss how the explosion in genomics based sequence analysis, allied with parallel developments in synthetic and molecular biology, have the potential to fast-track the discovery and subsequent improvement of a new generation of marine biocatalysts.

## 1. Introduction

With the oceanic ecosystem covering approximately 71% of the Earth’s surface, the marine environment represents the largest and most promising aquatic reservoir of biodiversity on the planet. The extent of this biodiversity, encompassing all orders of life, is already well established, making the marine spatial niche one of the most abundant sources of natural richness on the face of the planet [1,2,3]. The combination of species richness with the unique physicochemical properties offered by the marine ecosystem (such as high osmolarity, broad range of different temperatures, and different pH), allied with the extent of uncommon functional groups found in this environment (e.g., isonitrile, dichloroimine, isocyanate, and halogenated functional groups), underpins the breadth of chemical biodiversity in this biotechnologically important niche. Increasingly, there is growing evidence of the promising potential of untapped bioactive compounds isolated from the marine ecosystem with pharmaceutical and biotechnological applications [4].

In terms of phylogenetic and functional diversity, recent advances in molecular ecology, metagenomic and ecological modelling depict microbial organisms as representing the most relevant biological group in the marine environment. Estimated at 10^4^ to 10^6^ cells per milliliter, microbial biomass combined with environmental complexity and high turnover rates underpins the genetic diversity of the oceanic microbiome [5]. Marine bacteria in particular have been reported to produce a diverse array of secondary metabolites, with a proven benefit in human health [6,7]. More recently there has been increasing interest in bioprospecting the marine environment for biocatalytics, not least due to the likely presence of unique substrates within this ecosystem. By definition, biocatalysts are enzymes from natural sources that modify the rate of a particular reaction. These can range from classical enzymes such as lipases, proteases and nitrilases, to the modular enzymes involved in the synthesis of complex natural products such as polyketide synthases (PKS) and Non-Ribosomal Peptide Synthases (NRPSs). This latter class of biocatalyst has received considerable attention in light of the considerable interest in natural products and their potential for chiral resolution. The physicochemical properties required for many industrial transformations are consistent with those prevailing in the oceans, promising compatibility with existing industrial processes [8,9]. The use of novel biocatalysts is not only driven by their high versatility, but also owing to, the relevant intrinsic enzyme-associated features such as, regio-, chemo-, and enantioselectivity, as well as, the clean and cost-effective mechanisms that are represented by enzyme-catalyzed reactions [10]. These features, when implemented in industrial processes, contribute to a reduction in toxic effluents and can markedly reduce costs [11]. Generally encoded as single transcripts, the transformation of natural and non-natural compounds by microbially-derived enzymes (biocatalysis) represents a highly efficient and fruitful strategy applied for the production of many valuable compounds within biotechnological and pharmaceutical industries [10,12,13,14].

Current estimates put the market for biocatalysts at approximately US$4 billion [15] and the extent of enzyme categories employed continues to expand. Nitrilases, proteases (used in detergents and in pharmaceutical and chemical industries to degrade proteins into amino acids), lipases (synthesis of fine chemicals), transaminases, and glycosyl hydrolases such as cellulases and xylanases (paper and pulp industries) are among the most sought after activities. Many of these activities also support conversion to biofuels, with glycosyl hydrolases particularly important in this regard. Therefore, there is keen interest in developing biocatalysts for application in a diverse spectrum of industrial processes. One aspect to that has been the biodiscovery of novel biocatalysts from environmental samples using genomics based technologies. By virtue of their ability to inhabit and persist in extreme and severe environments (e.g., glaciers [16]; Arctic soil [17] and deep-sea hyper-saline brine pools [18]), the potential for microbes to deliver on novel biocatalytic functionality is considerable [19,20]. Marine niches, although still largely unexplored, have already delivered abundant diversity of novel microorganisms with unique functions [21]. In this regard, the extent to which they encode novel biocatalytic activities would be a key determinant in the “hit rate” of prospective mining studies.

**Figure 1:**
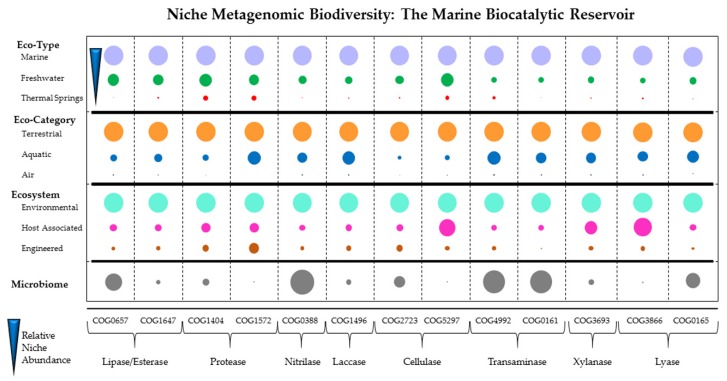
Relative niche abundance distribution based on clusters of orthologous groups (COGs) analysis of main enzymes with interesting biotechnological applications. The diameter of the spheres depict the relative abundance of COGs analyzed as the gene count/metagenome count ratio. At the microbiome level, the size of the different COGs are represented in relation to the most abundance COG (Nitrilase). At the three specific levels analyzed (Ecosystem, Eco-Category and Eco-Type), the size of the different circles are represented in relation to the most abundant COG per level. At Ecosystem level, the highest relative abundance of the different COGs belongs to the Environmental Ecosystem (light blue). At Eco-Category level, the most abundant Ecosystem is Terrestrial followed by Aquatic. Within Aquatic, the highest relative abundance of the different COGs belongs to the Marine Eco-Type (light purple) followed by Freshwater (green), supporting the biotechnological potential of the marine as an alternative source of biocatalytic activity to the well explored terrestrial datasets.

A simple representative COG (clusters of orthologous groups) analysis of the available metagenome datasets (IMG Database) reveals the marine ecotype as an abundant source of protease, lipase, cellulase, transaminase and other industrially relevant biocatalytic activities, underpinning the importance of this resource (Figure 1). A more comprehensive COG search of other interesting enzymes is included in Supplementary File 1. While the relative abundance of COGs was found to be higher in terrestrial ecosystems than in the aquatic environment, the terrestrial environment has already been extensively mined for similar activities. Focusing on the aquatic environment, marine metagenomic samples followed by freshwater samples represented the highest relative abundance of all eco-types for all the different COGs assessed. Of the COGs analysed, three were particularly abundant in the aquatic eco-category, namely COG1572 (protease), COG1496 (laccase) and COG4992 (transaminase). This fact, linked to the presence of unique substrates available in the marine environment, reinforces the promise of this diverse ecosystem as a source of novel biocatalysts. Of course, the relevance of this centres on what constitutes the “ideal biocatalyst” [22,23]. Parameters such as activity, efficacy, specificity and stability are key considerations in the selection of enzymes for different applications [24]. Enzymes with higher catalytic efficiency on insoluble substrates, increased stability at elevated temperature and at defined pH, and higher tolerance to end-product inhibition are particularly sought after [25,26]. Until recently, the “best hit” derived from a microbial source, either culturable or non-culturable would typically not have been a perfect fit with the industrial process or drug development pipeline. Downstream manipulation using molecular tools would typically have been employed to adapt the selected enzyme for the particular process requirements. With the advent of synthetic biology and the development of improved biodiscovery tools, the “ideal biocatalyst” has become more of an attainable goal than an aspiration. Key to this has been the explosion in genomic sequencing, the development of annotation tools, and the refinement of the bioprospectors toolkit. 

Thus, in the present review we discuss the current genomic approaches, including the identification, heterologous expression, and improvement of novel industrially relevant biocatalysts from the marine environment. Covering both culturable and non-culturable approaches, a comprehensive appraisal of the tools for genomics based biocatalysis is presented. Subsequently, the range of molecular tools used for biocatalyst improvement are discussed, covering areas such as directed evolution and synthetic biology.

## 2. Marine Environment as a Biocatalytic Reservoir

In the last decade, expeditions such as the Sorcerer II (2003–2010), the Malaspina (2010–2011), and the Tara Oceans (2009–2013) have profiled the taxonomic and metagenomic content of the marine ecosystem. Combining concerted global sampling efforts with high-capacity data analytics, we now have unprecedented access to the structure and functionality of the marine microbiome [5,27,28]. The breadth of microbial diversity encoded in the marine ecosystem is highlighted by the recent TARA ocean expedition, which predicted more than 40 million novel genes from their sampling programme [5]. Perhaps it is not surprising given that the marine ecosystem itself is subject to extreme differences in environmental conditions, even within the same geographical area. In this sense, from the Polar ocean and Polar coastal ecosystems, to the equatorial and hydrothermal equivalents, microbial adaptation to these extreme environments is likely to underscore bioactivities with new and commercially important properties. In this respect, of all of the organisms that inhabit extreme environments, microorganisms are best placed to thrive under severe conditions that are too harsh for animals, plants and other organisms too [29]. Extreme values of temperatures, pH, salinity, oxido-reduction-potential, and also combinations thereof, are successfully tolerated by species of marine microorganisms. According to Sarmiento and colleagues (2015), in many cases enzymes derived from extreme habitats have undergone an adaptive process to withstand these severe conditions, leading to changes in the secondary sequence and tertiary structure, flexibility, charge, and/or hydrophobicity [30]. Therefore, the ecological resilience of the marine microorganisms in these extreme environments may underpin their potential biotechnological applications. In light of the fact that the vast majority of general industrial processes are conducted under harsh condition such as, acidic or basic pHs, extremely low and high temperatures and elevated salinity, it is unsurprising that there has been increased interest in these so called “extremoenzymes” [4,31,32]. There are some examples of cold-adapted enzymes isolated from marine bacteria of great interest for diverse industrial markets, such us, technical enzymes used in molecular biology, food and beverage and detergents, amongst others. One example of a commercially available cold-adapted enzyme is the Antarctic Thermolabile UDG (New England Biolabs), a recombinant Uracil-DNA *N*-glycosylase produced in *E.coli* derived from a psychrophilic marine bacterium.

Of course, the genetic diversity and environmental complexity of the marine ecosystem in itself does not necessarily mean that valuable and new biocatalytic activities will be encoded therein. Indeed, the recent profiling of the marine microbiome performed by the Tara Oceans project reported that 73% of the core oceanic microbiome is shared with the human gut microbiome, in spite of the vastly different physicochemical properties of both ecosystems [5]. Furthermore, despite a broad range of fluctuating environmental parameters, temperature appears to be the dominant driver in shaping the microbiome composition, at least in the sunlit epilagic ocean layer in which many sponge associated communities exist [5]. However, it is important to consider that many of the novel and highly active natural products that have been isolated from marine organisms have come from low abundant and slow growing species. In fact, the role of the most abundant organisms within the microbiome is not yet clear and the most abundant groups may not be the most active ones [33]. It is estimated that marine species present double the chance of obtaining at least one gene in a patent than their terrestrial counterparts [34], while the success rate in finding novel active chemicals in marine organisms is 500-fold higher than that for terrestrial species [35]. Therefore, the marine ecosystem is certainly worth pursuing in the search for new and improved biocatalytics.

## 3. Biocatalysts as a Valuable Alternative to Traditional Chiral Chemical Synthesis

Thousands of natural chemical transformations are performed by diverse enzymes produced by living organisms as part of their natural physiology, enabling growth and persistence in their respective habitats. For some this can be the relatively hospitable nutrient rich environment of a compost heap or fertile soil. For others it can be extreme as with hydrothermal vents of the ocean presenting uniquely adapted enzymes for conversion of substrates to specific products [10]. Biocatalysts in general, and marine biocatalysts in particular, have several advantages over non-biological catalysts. The physicochemical properties, the presence of novel substrates and the breadth of diversity promised by genomic sequencing from the marine environment, underscores the inherent features of biocatalysts in general, such as their exquisitely precise chemo-, regio-, and stereocontrol. Moreover, catalysed reactions often proceed both under mild and neutral aqueous conditions, circumventing the need for toxic organic solvents or heavy-metal catalysts, and in addition, by virtue of enzyme selectivity, biocatalytic routes can preclude the need for synthetic protecting-group manipulations. However, perhaps one of the most important features of biocatalysts is their capacity for the synthesis or resolution of chiral molecules [36]**.** Through natural chirality of enzymes, most of the natural molecules having stereogenic centers, such as, carbohydrates, nucleosides, amino acids, proteins, alkaloids and hormones, are found in the single enantiomeric form. Thereby, nature creates and imposes stereoselectivity by means of enzymes, which are highly efficient biocatalysts [37].

**Figure 2:**
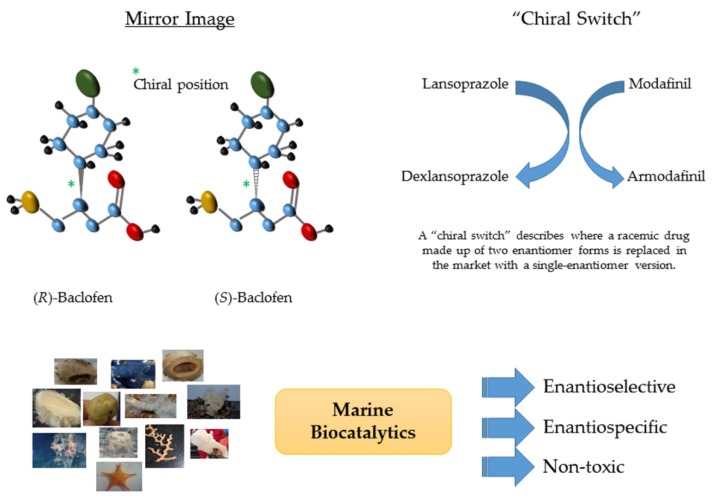
The potential of marine biocatalysts in chiral synthesis. (A) Chirality example: Baclofen. (*R*)-enantiomer is 100 times more effective than (*S*)-enantiomer. (B) Pharmaceuticals application coupled with chirals with depicted by two relevant cases of commercial drugs, Lansoprazole and Modafinil. (C) Features of marine biocatalysts that make them excellent candidates for chiral synthesis of fine chemicals.

In all the biological systems, chirality is a ubiquitous feature that plays a very important role in many and varied processes [38]. Chiral molecules exist when despite having identical composition, the components of the molecule are arranged in a non-superimposable mirror image composition, centred around an asymmetric carbon atom (Figure 2A). The two non-superimposable mirror images of a chiral molecule are called enantiomers. Enantiomers show practically identical physicochemical properties, and in many cases their biological activity can be similar. For example, (+)-Aeroplysinin-1 was the first brominated derivate from the marine sponge *Aplysina aerophoba* and the antibacterial activity of both stereoisomers was comparable [39]. However, this is not always the case and many enantiomers or racemic drugs exhibit different functionalities in biological systems [40]. One example of differences in the functionality of enantiomers is the case of Baclofen (Figure 2A). Baclofen (4-amino-3-*p*-chlorophenylbutyric acid) is a chemical analogue of an inhibitory neurotransmitter-γ-aminobutyric acid. Although marketed as a racemic mixture, the (*R*)-enantiomer of baclofen is 100 times more active than the (*S*)-enantiomer. Furthermore, only the *R*-enantiomer of baclofen is stereospecifically active at GAGAB-receptors [41]. In more extreme cases, toxic effects of a particular enantiomer can manifest, as seen with the classic example of thalidomide. Another example of this is salbutamol, best known as the active agent in inhalers. (*R*)-(−)-salbutamol (albuterol) is responsible for bronchodilator effects. while (*S*)-(+)-salbutamol has little bronchodilating activity, actually causes hyperkalemia, and has been implicated in eosinophil activation and pro-inflammatory properties [42]. Therefore, both in terms of safety and efficacy, chirality is now a crucial factor of many drug products and thus the production of single enantiomers of drug intermediates, as well as, drugs themselves, is becoming increasingly important in the pharmaceutical industry [43].

To date, the main target has been to promote the chiral separation of interesting enantiomers, as well as, the analysis of racemic drugs in the pharmacological area, avoiding or replacing enantiomers that could play a less effective or negative role [44]. In the pharmacology field, the majority of racemic drugs display an unequal activity of their enantiomers. This is the case for a number of important drugs such as anticoagulants, antibiotics, proton pump inhibitors amongst others that currently undergo chiral inversion or “chiral switch” to a more effective single-enantiomer version (Figure 2B). Recent single enantiomers introduced to the market replacing racemic mixtures have been (*S*)-Lansoprazole/(*R*)-Dexlansoprazole (a proton pump inhibitor) and (*S*)-Modafinil/(*R*)-Armodafinil (a dopamine uptake inhibitor). *S*-enantiomers have been replaced in the market for their *R*-versions due to their higher and more prolonged activity [44,45]. Similarly, chiral chemicals are also usually required as key intermediates for the synthesis of a variety of pharmaceuticals, agrochemicals, food ingredients, flavours, and fine chemicals [46].

Both, the breadth of use chiral synthesis and improvement of chiral separation methodologies, as well as, the publication of the FDA’s policy statement for the development of new chiral drugs on 1992 (http://www.fda.gov/Drugs) [47], had contributed considerably to develop single-enantiomers drugs by pharmaceutical manufacturers.

It is well established that conventional chemical synthesis routes to chiral products, are an expensive alternative, that typically require harsh reaction conditions (such as elevated temperature, high pressure, strongly basic or acidic conditions), which have a negative environmental impact. Biocatalysis promises a cleaner and more cost-effective process with excellent selectivity in asymmetric synthesis. Biocatalysis can be employed in the asymmetric total synthesis of groups of naturally occurring chemical compounds, combining the flexibility of chemical routes with the high degree of chemo and enantioselectivity displayed by enzymes. Therefore, the challenge facing the biocatalysis community is how to extract or mine the marine ecosystem for novel biocatalysts that will fit the industrial drug development pipeline and perform chiral conversions as required. In some cases, the marine enzymes already possess chiral activity, as reported with the synthesis of chiral hydroxy esters using Actinobacteria [48]. In other cases where the substrate profile of the enzyme is particularly interesting, directed evolution and random or targeted mutation of lead biocatalysts is an option (discussed later). Another area worth pursuing is the use of PKS enzymes and the different enzymatic domains that make up each modules as biocatalysts which enable the synthesis of polyketides *in vitro*. Hughes and colleagues reported how the terminal module and the thioesterase (TE) module of the erythromycin PKS (EryMod6EryTE) was capable of generating triketides on a scaled-up level from simple synthetic diketides and enzymatically generated building blocks [49]. Piasecki and co-workers demonstrated the ability of PKS enzymes to generate chiral building blocks on a preparative scale, going so far as to propose that “if PKS enzymes, themselves, were harnessed as biocatalysts, covered chiral building blocks and biologically active molecules would be more readily accessed” [50].

## 4. Culture Dependent and Independent Approaches to Unravel the Biocatalytic Potential of the Marine Environment

The marine ecosystem has been described for its great potential of novel bioactive compounds used in pharmaceutical and biotechnological industries [51,52,53,54,55,56,57,58]. Among the bioactive compounds produced by marine microorganisms, biocatalysts have emerged as an interesting alternative to the classic catalyst. In addition to their potential for chiral synthesis and their contribution to promoting green chemistry synthesis [59,60], marine biocatalysts display unique physicochemical properties, can transform spatial niche-unique substrates, and represent an untapped novel biodiversity not likely to be matched by terrestrial niches which have been extensively explored.

One consequence of the unique physicochemical and nutrient conditions of the various marine spatial niches is our inability to successfully cultivate the microbial diversity that exists therein. It is well known that isolation and cultivation of novel marine microorganisms is a major bottleneck for the discovery of novel marine bioactive compounds. Typically only from 0.001% to 1% of bacterial isolates can be recovered and grown under laboratory conditions [61]. In recent years, different approaches have been taken in order to improve the success rate of culturable bacteria [62], including the efforts of the currently active EU FP7 MaCuMBA consortium (http://www.macumbaproject.eu/) [63]. However, the fact that most microorganisms exist within polymicrobial communities in their natural environments, perhaps evolved to exploit interdependence with other microorganisms in these complex spatial niches, hampers efforts to culture microorganisms in artificial culture media [1].

**Figure 3:**
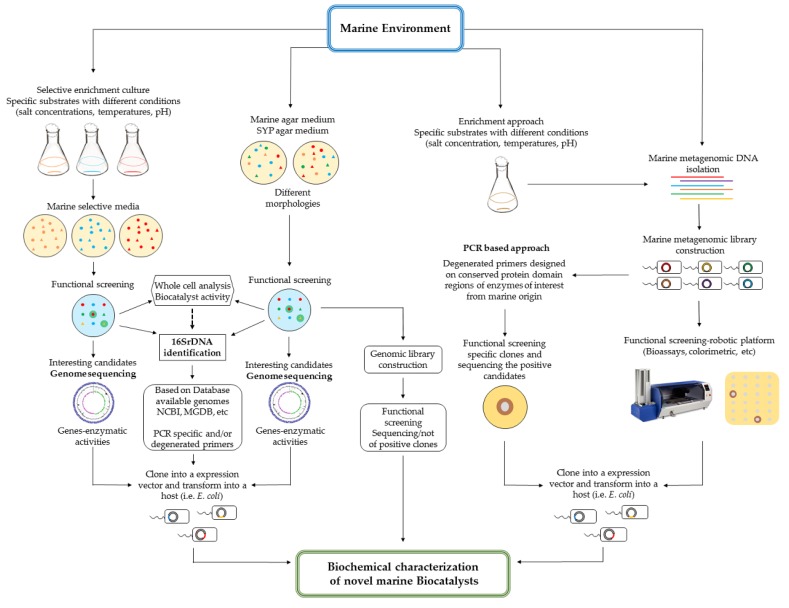
Schematic representation of the different methodologies followed to discover novel marine biocatalysts based on culture dependent and independent approaches.

Despite the low percentage of culturable bacteria, progress made in the last decade regarding Next Generation Sequencing (NGS) has generated a vast amount of genomic information that scientists can exploit in order to unravel the biotechnological potential of natural environments [64]. It is known that more than 70 bacterial phyla have no cultured representatives [1]. To explore this massive untapped genetic information, the latest advances in “omics” technologies are currently being used [65]. Metagenomics is one such approach, involving genomic and gene expression analysis of both culturable and unculturable microorganisms [66]. Based on genomic technologies (genome mining and metagenomics libraries, Figure 3) novel biocatalysts have already been described from marine microbial sources, primarily using three approaches (summarized in Table 1). Genome-based screening methodologies employ bioinformatic searches for specific protein domains in the genome sequences of marine culturable bacteria. These are limited by both the culturability of the marine organism and also the presence of an already known sequence or motif. Of course it is important to note that while an enzyme identified in this way will almost certainly possess the same catalytic motif as the already characterised subject upon which the search is based, the properties and substrate specificities of these enzymes have the potential to be remarkably different. Function-based screening is generally based on high-throughput analysis of metagenomics libraries (the culturable and unculturable biome) for specific biocatalytic activity, more often than not using generalised substrates. An example of this is the use of tributyrin for lipase screening. However, while this approach has the potential to bypass potential bottlenecks such as sequence divergence by focusing on functionality, its full potential is somewhat limited by the use of these generalised substrates. The real potential for the screening approach will be realised when challenging substrates, that are commercially important, are available and adapted for screens. This will make the search more challenging, but the outcome will be more fruitful. Sequence-based screening differs from the genome based approach described above in that it is based on the sequencing of metagenomic libraries. Similar to the genomic approach, specific bioinformatic searches are used to identify DNA sequences/protein domains of enzymes of interest. Alternatively, the genetic information can be extracted from the libraries using sequence-informed degenerate primers based on the enzymes sequences present in different databases. From a sequencing perspective, the potential biodiversity currently existing in publically available databases (between 10^4^ and 10^5^ protein sequences) is markedly lower than the mathematical potential of a fully random selection of amino acids (10^201^ for 200-amino acid proteins) [23]. This indicates a significant level of coding-based constraints, whereby not all mathematically possible sequence diversity can or do manifest in nature. This has consequences for downstream synthetic approaches used to optimize or improve novel biocatalysts, irrespective of their source. Mathematical models coupled with smart molecular methodologies will enable researchers to bypass incompatible sequence combinations, thus fast-tracking the creation of functionally viable derivatives. First, however, we have to be able to access that genomic data, and that requires us to access both the culturable and non-culturable organisms that encode the full breadth of sequence diversity.

### 4.1. Culture Dependent Approach

#### 4.1.1. Marine Bacteria as an Untapped Source of Novel Biocatalysts

The advent of molecular phylogenetic analysis tools such as 16S rDNA and ISPro sequencing has led to the realisation that a significant proportion of microbial species have never been cultured. It is estimated that somewhere between 10^5^ and 10^7^ distinct prokaryotic and lower eukaryotic species exist, with organisms from extreme environments proving the most refractive to conventional culturing techniques [100,101]. In recent years, relevant improvements have been performed in order to increase the number of culturable bacteria based on classical approaches, trying to mimic the natural conditions of the marine environment [102,103,104].

The marine environment harbors an enormous bacterial diversity, with microbes being described as the primary biomass producers, playing a key role in the global cycling of elements within this ecosystem [105]. This role is underpinned by the large repertoire of biocatalytic activities encoded within marine metagenomes, facilitating the biotransformation and cycling of marine-specific substrates that enable their colonisation of this niche. As such, marine microbes are continually exposed to extreme conditions found in the different sub-habitats, displaying a high biochemical diversity reflecting the presence of unique substrates. Marine microbes can exist as planktonic free cells or as sessile biofilms within polymicrobial communities, the latter promoting persistence in harsh ecological niches. Bacterial colonies are often compared to simplistic biofilms, with Kolter and Greenberg describing them as “air exposed biofilms” [106]. However, other studies have shown that bacterial colonies more closely resemble planktonic cells “stranded” on a surface, so therefore the line between both lifestyles is somewhat blurred in need of further clarification [107]. Marine microbes can also live in symbiosis with marine invertebrates, adding another layer of complexity to their interactome. Marine sponges have been reported to produce an array of diverse bioactive compounds, many of which we now know to be produced by the symbiotic bacteria [108]. The marine sponges themselves produce metabolic waste and different secondary metabolites including halogenated organic compounds [109,110]. These compounds can potentially play an important role as unique substrates that support the evolution of novel biocatalysts such as halogenases and dehalogenases [111,112]. The novelty attributed to these activities has been varied and ranges from sequence level, physicochemical, substrate specificity amongst other properties. While sequence novelty is of interest at the community level, there is a strong likelihood that not all of the sequence biodiversity observed in marine biocatalysts will manifest at the functional level. Glycosyl Hydrolases for example are classified using the CaZy system, based on sequence motifs rather than the classical enzyme classification. Novel GH sequences classified in this way will not necessarily perform new biotransformations or possess improved physicochemical properties. Therefore, functional characterization of enzymes possessing novel sequences needs to be fast-tracked to inform future studies. Notwithstanding this, as the number of biocatalysts harvested from the marine microbiome continues to expand, the possibility for new and improved functionalities becomes more of a reality.

The discovery of novel biocatalysts from culturable marine bacteria has been achieved through distinct methodologies and approaches. One of the most commonly utilised approaches from a culture dependent point of view is the detection of novel biocatalysts based on functional screening followed by PCR typing for taxonomic profiling. Once identified, individual genes encoding the required activity can be cloned, expressed in a suitable host and the purified protein characterized biochemically. Using this approach, several enzymes from marine microbes such as dehydrogenases, lyases, xylanases, chitinases, epoxide hydrolases, and esterases, between others, have been detected [113,114,115,116,117,118,119]. One of these examples is the case of cold-adapted isocitrate lyase (ICL) from the psychrophilic bacterium, *Colwellia psychrerythraea* [118]. An alpha-amylase from *Nocardiopsis* sp. (deep sea sediment of Prydz Bay) and a carboxymethyl cellulose from *Marinimicrobium* sp. LS-A18 were identified using both whole cell and crude extracts [120,121]. In the specific case of α-amylase the activity was determined by detecting the amount of reducing sugars liberated followed by thin layer chromatography (TLC) analysis to detect the hydrolysis products of starch [120]. Adding subsequent additional steps of protein purification, enzymes such as dehalogenase and alkaline lipase were detected [112,122]. The authors also demonstrated the presence of two distinct 2-haloacid dehalogenase activities detected in the cell crude extract after ammonium sulfate fractionation in the bacterial strain *Pseudomonas stutzeri* DEH130 isolated from the marine sponge *Hymeniacidon perlevis* [112]. Dehalogenase I was mainly active toward d-2-chloropropionate (d-2-CPA), whereas dehalogenase II exhibited at least 10 times more activity against l-2-chloropropionate-l-2-CPA. This suggested that *P. stutzeri* DEH130 could contain two stereo-specific dehalogenases. *Bacillus smithii* BTMS 11 strain isolated from marine sediment, was the source of a novel alkaline lipase, extracted by ammonium sulfate precipitation and ion exchange chromatography [122]. Enzyme characterization by SDS-PAGE and zymogram analysis confirmed the protein band which showed the lipase activity. Other approaches have been based on the construction of genomic libraries of bacterial candidates for a pre-determined enzymatic activity, from which a low temperature catalytic cellulase and two different pectate lyases isolated from the Antarctic bacterium *Pseudoalteromonas haloplanktis* have been identified [123,124]. Among the 10,000 clones screened for the cellulolytic activity using l-agar plates with carboxymethylcellulose and Trypan blue, only one positive clone was isolated [123]. In the case of pectate lyases, approximately 15,000 clones were screened in LB-agar plates containing 0.1% (w/v) citrus pectin and five colonies with pectinolytic activity were observed. The DNA was sequenced and two open reading frames were found, both ORFs exhibiting sequence identity to pectate lyases from *Erwinia chrysanthemi* [124].

Nowadays, more modern approaches due to development of the NGS technologies are considered. Genome mining could emerge as an alternative to the culture dependent approaches described above. Genome mining offers the possibility of discovering potentially novel biocatalysts using bioinformatic tools. Protein domain analysis of genome sequences from culturable organisms, could reveal potential novel biocatalysts, which cannot be detected by the previous experimental procedures mentioned. However, this approach is limited to near neighbor proteins, limiting the novelty somewhat to previously identified domains. This is due to the fact that the sequence based searches are based on similarity algorithms that score based on sequence identity. The more novel the sequence, the less likely a “hit” will present itself during the analysis stage.

#### 4.1.2. Genome Mining: An Under Exploited Source of Biocatalyst Discovery

The expansion of microbial genome sequencing over the last 20 years [125], through the increase of the speed of NGS and the decrease in cost of genome sequencing, has provided the basic tools for the *in silico* discovery of genes and gene clusters related to the production of novel marine natural products and biocatalysts [64]. However, the rapid increase of marine metagenomic DNA information in the database [126] has largely supplanted the directed genome approach. Recent efforts to decipher the extent of the oceans microbial biodiversity and the novelty of marine bioactives with biotechnological potential include the global Ocean Sampling Day; a simultaneous sampling campaign of the world’s oceans performed under the umbrella of the Micro B3 Project from the EU (https://www.microb3.eu/osd) [127]. To date, there are only few reports that describe the use of genome sequence of marine bacterial strains to identify and subsequent characterize potential biocatalysts [67,68,69]. 

In 2008, de Pascale and co-workers describe the presence of a novel cold-active lipase (called Lip1) from the Antarctic bacterium *Pseudoalteromonas haloplanktis* TAC125. These authors used the previously published genome sequence of this bacteria [128] to carry out a computational search and identification of lipase-encoding sequences. Based on conserved motif analysis and multi alignments of seventeen homologous genes, Lip1 and other related genes constituted a novel lipase family, typical of psychrophilic marine γ-proteobacteria. Another study carried out by Novak and co-authors (2013) in the psychrophilic bacteria *Psychromonas ingrahamii* DSM 17664 described for the first time the biochemical characterization of an L-haloacid dehalogenase. Based on the available genome sequence of this bacterial strain [129], the authors used specific primers designed on the sequence of the *pinHAD* gene to amplify and clone this gene into the pET-28a protein expression vector. This enzyme was over-expressed, purified using nickel affinity chromatography columns, and biochemically characterized. The activity was measured using a colorimetric assay, based on the presence of phenol red at pH 8.2, and using as the substrate monochloroacetic acid. Substrate specificity analysis showed that this enzyme presented highest activity towards substrates with short carbon chains (≤C3), and that it was stable in different organic solvents at different concentrations. Despite the fact that the enzyme was isolated from a psychrophilic bacterium, it showed an optimal activity temperature of 45 °C.

Essentially, a similar approach has been used by Li and co-workers (2013), to determine bioinformatically and subsequently, amplify and clone a NADPH-dependent aldehyde reductase in *Oceanospirillum* sp. MED92, based on its previous genome sequence report [130]. This enzyme called QsAR, showed activity against a wide range of aliphatic and aromatic aldehydes, but displayed no activity against ketones, suggesting that this enzyme catalyzed the chemoselective reduction of aldehydes in the presence of ketones.

The large number of marine bacterial genome sequences coming on stream in the publically available databases suggests that the genome mining approach will provide a lucrative window for the identification of novel biocatalysts, notwithstanding the near-neighbor constraints described above that hamper sequence homology based approaches.

### 4.2. Culture Independent Approach: Metagenomic for Biocatalyst Discovery

The culturable bottleneck has several aspects. It is generally accepted based on genomic data that the majority of marine prokaryotes have not yet been cultivated. Of even more concern is the fact that, of those organisms we can cultivate, the majority do not come from extreme environments, niches from where valuable activities are likely to be extracted [131]. Metagenomics provides a valuable tool to attain the genetic information of the vast majority of the microbial community in a sample, underpinning a major advance in understanding of the existing genetic diversity, population structure, and ecological roles of microorganisms in the marine environment. According to Handelsman, the term metagenome can be defined as “the genomes of the total microbiota found in nature”, referring to the sequence data directly sampled from the environment [132]. Thus, metagenomics provides culture independent access to valuable genetic resources of the uncultured microbes [77]. With the upsurge in the development of more efficient and powerful tools to explore the huge genetic potential that comes from natural environmental sources, the modern biotechnology field is well placed to deliver on the early promise of novel bioactivities [66,78,132,133,134,135,136,137,138].

Metagenomics-based approaches have focused on both gene clusters and singular genes encoding enzymes, delivering on the enhanced discovery of biocatalysts for synthesis and production of secondary metabolites and other bioactive compounds [139]. Barone and colleagues (2014) categorized the three distinct environments for which metagenomics-based biodiscovery can be applied: highly diverse environments like soil and seawater, naturally or artificially enriched environments, and finally, extreme environments [1]. The breadth and diversity of the marine ecosystem encompasses elements of all three categories, providing unique habitats for the organisms living therein [140]. Marine microbes comprise a wide variety of communities that are well adapted to living in this challenging ecosystem, either surviving as free-living organisms or in association with other organisms such as marine animals [141]. Of course, harnessing such diversity is not without its own bottlenecks, even at the genetic level. The hit rate in metagenomics studies is both determined and limited by several factors: the chassis or host organism, the size and complexity of the target gene or gene cluster, its abundance within the sample, the screening method, and the fidelity and downstream functionality of the protein in the heterologous host. In this regard, the development of compatible broad host range shuttle vectors and suitable chassis organisms for expression and production of heterologous proteins remains a significant challenge. Furthermore, the reliance on classical screens has limited the extraction of novel activities even when these challenges are overcome in a particular sample. Therefore, an integrated and highly focused multidisciplinary effort will be required at all levels of the metagenomics pipeline in order to pursue the ultimate goal of harnessing the complete biodiverse potential of the marine and other ecosystems.

#### 4.2.1. Metagenomic Screening Strategies

As a rule of thumb, in all metagenomic screening strategies, once environmental DNA has been isolated, the first step is the construction of a DNA metagenomic library in a suitable host cell [142,143]. In itself, DNA isolation and packaging has proven a significant limitation to biodiscovery from rare-producing organisms due in part to low abundance, prohibitively high or low %GC content, as well as incompatibility with the cloning and vector systems employed. In some cases enrichment prior to DNA isolation can improve the hit rate, as in the case of symbiotic bacteria associated with marine sponges or other invertebrates [144,145,146,147]. Once the DNA has been captured, the success of the subsequent metagenomic screening strategies, and consequently the discovery of novel biocatalyst candidates isolated from unculturable marine sources, is directly determined by the degree to which gene expression and proper protein folding is achieved in the heterologous cell host [148]. Once the metagenomic library has been constructed, the metagenomic screening approaches comprises principally of two complementary methodologies that can be based on either function (activity) or sequence [149]. Function based screening, while direct and relatively informative, is hampered by the biased and often unsuccessful expression of foreign DNA in the heterologous host of choice, classically restricted to *Escherichia coli* (*E. coli*). On the other hand, while sequence based screening has benefited from the explosion in metagenomic sequencing projects, and the availability of sequence data for PCR or hybridization design, this approach is largely restricted to near neighbour sequences and may not deliver on the rare biocatalytic activities being sought from the marine environment. Nonetheless, both approaches are central components in the bioprospecting toolkit and advances in both approaches have greatly improved their utility.

#### Metagenomic Functional Screening

Metagenomic functional screening is based on the direct screening of potential function, before any analysis of nucleotide, ribonucleotide or protein sequences [150]. Thus, in order to detect efficiently the potential candidates (clones harbouring biosynthetic genes of interest and which are able to exhibit different modified phenotypes) several functional screens can be implemented. In terms of detection, the majority of these screens are a direct method where products of individual metagenomic clones can be detected either visually or spectrophotometrically [141]. Essentially, three classes of screens are employed, with the design and implementation specific to the chosen target activity. Enzyme activities are generally screened on bioassay Q-tray plates on agar supplemented with substrate for which conversion to product results in either a colorimetric change, a halo or zone of clearance, or alternatively a growth no growth phenotype. All three are easily scorable and the validation rate from these hits can be quite high. In liquid based screens, cells or cell lysates are used in multi-well (typically 96-well or 384-well) plates and the conversion of substrate to product can again be measured by growth no growth, or alternatively a spectrophotometrically measurable change in absorbance. Finally, using molecular tools and an increasing knowledge of the regulatory systems underpinning biocatalysis in living organisms, reporter fusions can be developed for use as “gene traps”, where conversion of substrate to product causes a regulatory protein to activate a fused promoter resulting in fluorescence, X-gal hydrolysis, or antibiotic resistance. The selection of a particular screening methodology can be enzyme specific, and some screens have been used widely within the biodiscovery community. There are many examples in the literature, extendable to a wide range of enzymatic activities, where function-based screening methods represent a valuable option, which have previously been employed successfully (see Table 1).

Lipases and esterases plays a prominent role as biocatalysts, being widely employed in many diverse fields such as textile, food, laundry, paper and pulp industries, biodiesel production and in the synthesis of fine chemicals [1]. In 2008, Chu and co-workers reported the identification of two novel esterases isolated from a marine metagenomic library derived from South China Sea [77]. In this study, positive candidate clones with lipolytic activity were detected by the formation of a clear halo around the colony growth after 2 days at 37 °C on LB agar plates supplemented with 1% (v/v) tributyrin and 1% gumarabic as substrates. Similarly, other authors identified the Red Sea Atlantis II esterase (EstATII) by means the same functional screening (after 3 days incubation, the appearance of a clear halo around transformant was indicative of a positive candidate with lipolytic activity), before further sequencing and identification of lipolytic encoding genes [84]. Similarly, several other independent studies on different metagenomic libraries from brine, the seawater interface of the Urania hypersaline basin, the Arctic sediment metagenome, a deep-sea sediment metagenome, and also from the marine sponge *Haliclona simulans*, described lipase and esterase activities. [76,78,95,96]. In all these cases, the different novel activities identified (five different esterases, two esterases with potential application, one cold-active lipase and one halo tolerant lipase) were identified after plating on a selective tributyrin or tricaprylin medium, according to their capacity to form clear halos. 

Other examples of efficient screening methodologies relate to the identification of glycosyl hydrolase activities, such as that described by Wierzbicka-Woś and co-worker (2013), where a novel cold-active glycoside hydrolase (BglMKg) was identified [90]. In this case the positive β-galactosidase candidates were selected by their capacity to hydrolyse X-gal, and consequently turned to dark blue. In this work, the authors also examined the enzymatic specificity of BglMKg towards different chromogenic substrates. A similar approach has been used by Lee and colleagues (2007) and Prabavathi and colleagues (2012), where the isolation of two novel protease activities was reported from marine metagenomic libraries derived from sea sediment [98,99]. In both cases, the screening of the metagenomic library for proteolytic activity was performed on SMA (skimmed milk agar: LB agar + 10% (*w/v*) milk solution with to a final concentration of 1% (*w/v*)) and the positive proteolytic clones were selected based on the formation of halo zone of clearance around the colony. As discussed earlier, the list of screening methodologies available for the isolation of biocatalysts from metagenomic libraries is well established and does provide a considerable hit-rate from marine and other source samples. However, many of the activities that have been isolated using these screens are not novel, nor does the screen assess or select for novelty by design. Therefore, the challenge remains to begin to design smart screens based on industrially relevant substrates to look for characteristics such as substrate specificity, selectivity, chirality and enzyme stability, the very features we are seeking in the “ideal biocatalyst”. That is not to say that generic substrates do not have a role in the functional screening approach. There is some evidence to suggest that enzyme promiscuity may provide access to novel activities, albeit not in a selective manner. Low efficiency but relatively unrelated enzymes may be detected in sensitive screening assays, as seen with the isolation of an esterase/β-lactamase dual activity from a leachate metagenomic library [151]. The key to this is recognizing and interpreting the outputs from the comprehensive primary screen, and selecting the appropriate downstream biochemical tests to validate the activity.

#### Metagenomic Sequence-Based Screening

The Metagenomic PCR-based approach (also called Metagenomic DNA Sequence-based Screening) is another alternative methodology, primarily based on the analysis of nucleotide, ribonucleotide or protein sequence. Typically, a set of degenerate primers are designed based on available consensus sequence data for the target locus are used. Alternatively, hybridization with probes can be used to extract the sought after genetic information. Either way, screening based on the detection of DNA and has the advantage of identifying novel activities without the need for expression in a heterologous host [142]. Of course, functional expression will still be required and present its own challenges. However, these limitations are avoided at the screening stage, reducing the rate of false negatives owing to inefficient heterologous expression. Depending on the depth of sequencing, analysis of metagenomic DNA environmental samples can provide a fundamental understanding of the ecology and biogeochemistry of uncultured marine microbes. From 2009 to 2015 the number of metagenome sequencing projects has increased from 199 to 607, while there are currently 22,455 non-metagenomic studies listed on the GOLD site (http://www.genomesonline.org) [152]. The enormous quantity of sequencing data presents an opportunity to explore the novel genes of interest that undoubtedly will exist therein. Nevertheless, there are limitations and this approach, not least the degree of novelty accessible through this approach. The fact that the method relies on known sequences deposited in databanks, it is restricted to the detection of homologous enzymes. To date, the amino acid identities of newly discovered sequences compared to those already present in the databases has been above 50%, and typically has been in the range of 70%–90%, with the possible exception of members of the lipase class of enzyme [153]. Therefore, extending below that range may require alternative approaches such as functional screening, or improved annotation and predictive software to identify domain structures.

Sequence analysis of environmental DNA from the Sargasso Sea Whole Genome Sequence (WGS) data performed by Cottrell and co-workers in 2005, enabled the search for novel hydrolases (CelM) used by Cytophaga-like bacteria, revealing the cellulase gene *celM* [91]. In this study, PCR primers were designed for the most abundant type of endogluconase identified in the WGS data set, and were used to screen a fosmid library constructed with prokaryotic DNA derived from the western Arctic Ocean. Jiang and co-workers (2010) constructed a plasmid metagenomic library from marine water samples derived from the Arctic Ocean [88], from which they identified the novel fumarase gene *fumF*. Fumarase activities have been extensively applied for the industrial conversion from fumarate to L-malate, and are another example of the breadth of profitable biocatalysts that can be mined from environmental samples. Wang and co-workers (2011) discovered a novel glycoside hydrolase activity (GH-57) from a metagenomic fosmid library derived from the Mothra hydrothermal vent and randomly pyrosequenced [89]. Using the fosmid library as a template, and based on DNA sequence analysis of the *gh-57* fragment, degenerate primers were designed to successfully amplify the *gh-57* gene fragment, which could subsequently be expressed heterologously. In another study, Fang and co-authors (2011) used the sequencing strategy to discover new bacterial laccase genes of 1.32 kb isolated from microbial metagenomic libraries derived from the South China Sea [92]. Their screen was based on the conserved region of laccase encoded copper-bing sites. Laccases, as a biocatalyst, have received attention for relevant applications in both environmental and industrial biotechnology. In spite of the obvious drawbacks in sequence and homology based searches, novel lipases have been isolated with greater frequency than other classes of enzymes from metagenomic samples. Indeed, new families of lipase have been described based on accumulating sequences and this extends the capacity to mine with greater depth for similarly novel sequences from marine and other niches [154]. 

As the extent of annotated diversity increases, so does our capacity to recognize and identify sequences with greater novelty through homology based searches. However, crucially, whether or not this translates into functional novelty, or indeed retains the annotated activity needs to be established post-screening. The challenge to the biodiscovery community is to accelerate the translation of sequence novelty into functional novelty. While it is clear that current screening approaches have led to the isolation and characterization of novel sequences, most notably with the lipase class, this has not as yet manifested as novel functionality. Assay development and more sophisticated annotation programmes can assist in achieving this goal. Alternatively, the novel sequences obtained from metagenomic samples can be used as platforms for a series of downstream molecular manipulations, collectively described as directed evolution.

## 5. Biocatalytic Improvement by *Directed Evolution*

As mentioned in the previous section, the rapid increase in NGS technologies, has provided an immense amount of novel biocatalyst sequences from different sources [155]. Despite the fact that biocatalysts potentially exhibit significant advantages when compared with chemical catalysts, in many cases natural enzymes are not functionally optimized for practical application in the industrial sector. In this respect, the directed evolution of enzymes has been described as a powerful tool for improving the critical traits of biocatalysts [156,157,158,159].

### 5.1. Directed Evolution of Enzymes

The marine environment could play an important role in the discovery of novel enzymes mainly due to the presence of novel substrates that cannot be found in other environments. Although a diverse range of marine enzymes have been discovered in the last decade, to date, there are a limited number of reports where directed evolution methods have been applied to improve the activity of marine derived primary enzymes [160,161,162,163,164,165,166]. Of course, questions as to the utility of directed evolution versus the search for rare and naturally adapted enzymes needs to be considered. As directed evolution technologies adapt from random modification to targeted structure based approaches, the stage at which molecular systems can provide the entire spectrum of possible sequences is approaching. At that point, the utility of sequence based searches based on known motifs may become redundant. However, for now, the tandem use of both approaches is most likely to yield the maximum success. In this section, we describe the implementation of directed evolution approaches that have been successfully applied to “improve” marine enzymes, and discuss how this can help unravel the huge potential promised by the diversity of the marine ecosystem. The sequence-diversity of marine biocatalysts, and their uniqueness relative to bioactivities from other environmental spatial niches, presents a new building block or scaffold upon which directed evolution approaches can be applied. This can potentially increase the possibility of creating biocatalysts with new and superior properties relative to those created using the more conserved scaffold sequences that have already been used.

**Figure 4:**
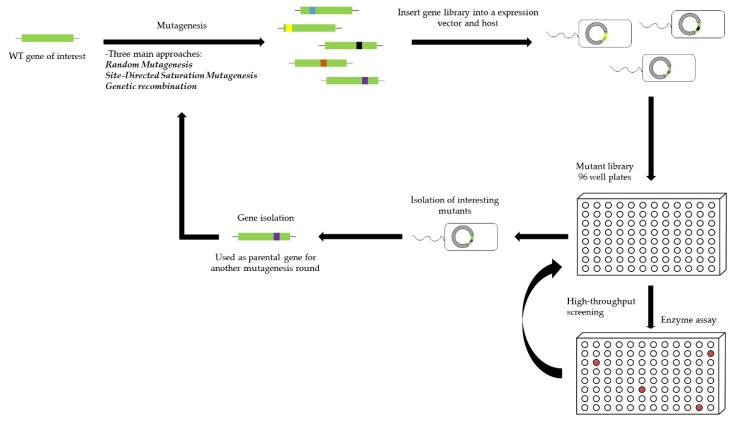
General overview of the different methods used in directed evolution involved in the library mutants construction.

The common element shared by directed evolution approaches, and also one of the major bottlenecks to this technology, is the generation of a diverse mutant library. Different approaches have been developed, using different mutagenesis methods to generate smaller and higher quality libraries [156,167]. Basically, three main strategies are followed for the generation of a sequence library: (1) random mutagenesis, (2) site-directed saturation mutagenesis, and (3) genetic recombination. A general overview of the basic workflow on directed evolution of enzymes is represented in Figure 4.

#### 5.1.1. Random Mutagenesis

The random mutagenesis approach is considered the classical mutagenesis technology for generating libraries, and is still broadly used. This approach only requires limited knowledge of the parent protein, and large libraries with low mutation rates and uneven mutational spectra are often obtained [167]. Different types of random mutagenesis have been described such as chemical mutagenesis, mutator strains and error-prone PCR (epPCR).

Chemical mutagenesis is the most traditional way for generation of mutant clones, and different chemical agents can be used for this purpose. Ethyl methanesulfonate (EMS) is one of the most common chemical agents utilized but also another compounds such as nitrous acid and bisulfite have been successfully used [168,169]. Recently, EMS along with other chemical and physical mutagens (nitrous acid, acrylamide, ethidium bromide and ultraviolet radiation) was successfully applied to improve an α-amylase activity from *Brevibacillus borostelensis* R1 isolated from marine water [163]. Although the increase in the fold yield of α-amylase activity was modest, reaching 1.41 as a maximum, at least nine mutants obtained by different mutagens were identified, providing new platforms for the next cycle of enhancement. Alternatively, it is well known that DNA polymerase during DNA replication can introduce mutations at different rates depending on the organism. Using mutator strains that contain non activated proofreading and repair enzymes such as *mutS*, *mutT* and *mutD*, DNA polymerase can increase the mutation rate [170,171]. The main problem associated with this approach is that the mutations not only affect the target gene selected for the library construction, but also introduce mutations that can be deleterious in the host genome. The drawbacks inherent in both strategies has led to the development of an *in vitro* strategy based on a PCR. Error-prone PCR (epPCR) described for the first time by Leung *et al.* 1989, uses a *Taq* DNA polymerase with no proofreading activity that is modulated by the composition of the reaction buffer [172]. In this context, the low fidelity of DNA polymerase generates random mutations during the PCR amplification of the selected gene. Thus, during each PCR cycle, DNA polymerase can increase the number of mutations per clone. A novel cold-active esterase from *Serratia* sp. isolated from marine environment, that showed a remarkable catalytic activity at low temperature, showed increased thermostability following epPCR cycling [166]. A random library of 8000 mutant clones was analysed based on retention of esterase activity after incubation at high temperature. One clone that showed higher thermostability was found and designated as 1-D5. Gene sequencing revealed three alterations in the amino acid sequence. Following this same approach, Zhou and colleagues (2015), increased the thermostability of an α-glucosidase from *Thermus thermophiles* TC11[164]. After the screening of 2,700 ep-PCR library clones, one mutant that carried one amino acid substitution (Q10Y) showed increased thermostability. Altough the residual activity was 20% higher in the mutant than in the wild type, the specific hydrolytic activity towards *p*NP-α-d-glucopyranoside (*p*NPG), was lost with the increase in thermostability. Furthermore, Chen and co-workers [161], used epPCR on the thermophilic cellulose *cel5A* gene from *Thermotoga maritima*, generating two mutants that had increased activity on pretreated switchgrass, reducing the recalcitrance of lignocellulosic biomass. A common thread to these approaches is the need for large libraries of clones, with low hit rates from the mutants. Furthermore, as the libraries generated by the random mutagenesis approach are larger and can be of limited quality, other focused strategies have been designed.

#### 5.1.2. Site-Directed Saturation Mutagenesis

Site-directed saturation mutagenesis (SSM) is a more focused approach, and can be used to create “smarter” libraries [157,173]. To date, many proteins are really well characterized structurally and the specific residues to bind different substrates or those involved in catalysis are well known. Although SSM can be applied to generate mutations in every position in a gene, previous structural data or homology based sequences are used to select specific residues [174]. In this approach synthetic DNA primers that carry the desired mutations are used in a PCR to test all 20 amino acids in a target position. This can be achieved using the trinucleotide NNN in the target amino acid. A small variation of this strategy is performed using the codon NNK, instead of NNN, decreasing the number of variants, and thus, reducing the screening efforts in the libraries generated [175]. Enzyme promiscuity has been considered as an important prerequisite for fast divergent evolution of biocatalysts. In this regard, the phosphotriesterase-like lactonase (PLL) enzymes exhibit classical lactonase and promiscuous phosphotriesterase activities. Theses enzymes are important as bioremediation tools for the degradation of neurotoxic organophosphates, being an attractive target for directed evolution techniques. Zhang and co-workers (2012) utilized a thermostable PLL from *Geobacillus kaustophilus* HTA426, isolated from marine sediments, as a parental gene [160]. Using a combination of SSM and epPCR, several active mutant variants were obtained. The most active mutant variant, 26A8C, that had undergone eight amino acid substitutions, showed a 232-fold increase activity against the OP pesticide *ethyl*-paraxon, and 737-fold decrease in lactonase activity. High hydrolytic activities were also shown for a wide range of OP pesticides. Zhang and co-workers(2015), utilized the same enzyme from the same organism to investigate the promiscuity of the binding site [165]. Based on amino acid sequence alignments with a phosphotriesterase enzyme (PTE) using also the crystal structure of the PLL from *G. kaustophilus* HTA426, the authors observed how the position Tyr99 (Y99) in the binding site of this enzyme, could be involved in the substrate discrimination. Using an SSM approach, they found one mutant with the change Y99L, that showed an 11-fold increase phosphotriesterase activity, and 15-fold decrease lactonase activity. A structural analysis of this mutant position revealed an outward shift of the adjacent loop 7 that was proposed to increase the flexibility of the active site, suggesting that the active site conformation loop regulates the promiscuous activity. Reetz and Carballeira (2007), proposed the Iterative Saturation Mutagenesis (ISM) where the authors combined in an iterative manner, the SSM approach described previously [176]. This strategy has been also used to successfully improve different properties such as thermostability, substrate or enantioselectivity [177]. For example, ISM has been successfully used to enhance the enantioselectivity of an esterase from *Bacillus stearothermophilus* [178], and to improve the activity of a Baeyer–Villiger monooxygenase [179]. Another approach reported by Reetz and co-workers (2005) called combinatorial active-site saturation test (CAST), is based on the fact that different amino acids positions can be simultaneously mutated, and this can affect the synergistic conformational and electrostatic activity of the enzyme [180]. Sandström and co-workers (2012), developed a novel method for radically reshaping the active site of a lipase A from *Candida antarctica* based on CAST library technology improving the activity and enantioselectivity toward difficult substrates [181].

#### 5.1.3. Genetic Recombination

DNA shuffling as a directed evolution method was described for the first time by Stemmer (1994) [182]. It is an *in vitro* DNA recombination strategy in which homologous genes (parent genes) from different species are randomly fragmented by DNase I and fragments of desired size are randomly reassembled using a PCR reaction without added primers. After this reassembly, PCR amplification with primers is used to generate full length chimeras suitable for cloning into an expression vector. This mutagenesis technique was successfully applied to create an *E. coli* aminotransferase that accepted β-branched substrates, which were poorly accepted by the wild type enzyme [183]. In another study [162], the authors addressed the bioremediation problem of herbicides in the agricultural sector. Glyphosate is a broad spectrum herbicide widely used in agriculture, and the glycine oxidase, has been described to catalyse the C–N bond in glyphosate. epPCR combined with DNA shuffling improved the activity of a glycine oxidase from a marine strain of *Bacillus cereus*. The most active mutant variant obtained in this study showed a 160-fold increase in substrate affinity and a 326-fold enhancement in the catalytic activity towards glyphosate. Through structure modelling and molecular docking, the authors suggested that the mutant position 51 (Arg51) close to the active site could play a role in the stabilization of the glyphosate binding.

This method accelerated greatly the evolution rate of these genes, but, only a small number of variants can be obtained when the parent genes present low sequence homology regions. In this respect, a related method reported by Coco and co-workers (2001) improved the recombination sequence and offered a more comprehensive exploitation of sequence space. This method called random chimeragenesis on transient templates (RACHITT) uses a similar approach to DNase fragmentation but utilizes a different way of DNA reassembly [184]. Although the new stream in directed evolution is focused on the use of sophisticated and more precise methods to generate smart libraries (small and high quality), these approaches require a deep knowledge in molecular structures, homology modelling and phylogenetic information to decipher which specific mutations are the most important to increase the activity of a determined enzyme. Random mutagenesis can overcome this requirement when these data are not available, and has the added advantage of creating mutations independent of what would be considered logical based on existing protein sequence information, and completely independent of those present through the normal evolutionary process. Fortunately, all the different strategies (random, SSM and genetic recombination) can be applied together, with the potential to maximize the likelihood of success [185].

## 6. Synthetic Biology

Despite the undoubted potential of metagenomics based biodiscovery for the isolation of novel biocatalysts, several issues need to be resolved before we can begin to realize the true potential of these technologies and harness the true extent of the natural biodiversity that exists in our environmental samples. These include (i) the bias encountered in using a heterologous host, typically *E. coli* for expression of metagenomic DNA, (ii) the ability of the vector system to express the target sequence, (iii) the capacity for the host to properly fold and potentially secrete the active protein, and (iv) the effectiveness and sensitivity of the screening strategy to detect the biotransformation when it occurs. A series of technological advances under the umbrella term of synthetic biology are under development to address each of these issues. Synthetic biology in its own right has a projected global market value of US$10 Bn for 2016 and is intrinsically linked to the directed improvement of industrially relevant bioactivities [186]. Comprising both enabling and core technologies, the area of synthetic biology will continue to grow and lead the genomics-based optimization of bioactive compounds for the foreseeable future.

It is perhaps unsurprising that the extent of natural biodiversity encoded in the earth’s microbiome has not yet manifested in screening or targeted biodiscovery programmes, given the focus on a limited spectrum of heterologous hosts or chassis organisms. For the most part, *E. coli* has been the organism of choice for synthetic biology, with *Streptomyces*, *Pseudomonas*, and *Rhizobia* being explored as alternatives. In spite of the availability of engineered *E. coli* host strains for optimized production of foreign DNA, the current limitations are borne out by screening of the same cosmid libraries in different hosts [187,188]. Therefore, performing the metagenomics based screening in these alternative hosts will almost certainly expand the range of expressed and detectable activities. This brings with it its own challenges with these alternative hosts being significantly less well adapted for heterologous expression than *E. coli* which has undergone decades of development and optimization for this purpose. However, potential chassis organisms such as *Streptomyces* and *Pseudomonas* do have the advantage of encoding vast repertoires of bioactive enzymes and secondary metabolites, thus possessing the machinery and metabolic systems to effectively express these activities. Increased understanding from molecular studies of the stress adapted phenotypes exhibited by these species will also underpin their development and optimization as chassis for metagenomics based expression.

Similarly, the design and integration of broad host and host-tailored vector systems for expression of heterologous DNA is a major challenge that has seen incremental advances in recent years. Owing to the fact that most biocatalysts are encoded as single genes, small insert expression libraries are particularly suitable for mining these activities from metagenomic samples, with the caveat that larger libraries are required for coverage. An additional advantage to this approach is the fact that genes are more likely to fall under the control of the strong plasmid promoter ensuring expression of the transcript. While important in their own right, it is the integration of both chassis and vector development into tailored natural product factories that will bring the most valuable advances in the area of biocatalytics and bioprospecting in general.

A key aspect of synthetic biology is the reprogramming and rewiring of regulatory systems in chassis organisms. Genomics based discovery of biocatalytic enzymes has highlighted the co-occurrence of genes encoding transcriptional regulatory proteins, unsurprising given that many of these activities are part of the core metabolic functionality of the microbial cell. The fact that these activities are programmable through either activation or repression lends itself to the artificial manipulation of transcriptional, translational, and post-translational regulatory systems in the optimal chassis organism. A key regulator of biocatalysis in microbial organisms is the LysR-Type Transcriptional Regulator (LTTR) family. We have previously characterized LTTR evolution among the Pseudomonadaceae, identifying several clades of co-evolved homologues [189]. Other transcriptional regulator families also contribute to the biocatalytic regulatory framework in the cell and understanding their role will facilitate improved chassis design. Additional synthetic biology approaches have been to engineer the ribosome of the chassis organism, engineer factors related to translational machinery in the host, provide plasmid encoded sigma factors and tRNAs that recognize rare codons, or alternatively to synthetically design codon-adapted genes for expression in the chosen heterologous host [190].

In spite of the developments in this area in recent years, several aspects have been identified as critical knowledge gaps in methods and technology (genome-scale engineering tools, DNA synthesis and assembly, analytical tools), biological platforms (biological design principles, genetically tractable organisms/chassis, minimal cell and *in vitro* systems) and computational tools and bioinformatics (information standards, databases) [186]. Further challenges are presented on an ongoing basis by changes in regulatory frameworks across different areas of governance for what is a global technology operating within a global biodiscovery sampling site. New directives such as the Nagoya protocol (https://www.cbd.int/abs/) [191] have significant implications for the use and manipulation of genomic data that need to be addressed within the framework of current and future biodiscovery programmes. Essentially, the Nagoya protocol governs utilisation of genetic resources for benefit, defined as “research and development on the genetic and/or biochemical composition of genetic resources, including through the application of biotechnology” making it subject to benefit sharing with the sovereignty in which it is discovered. Researchers globally will need to conform to this and other new directives in planning biodiscovery programmes and in developing the outputs for commercial benefit.

## 7. Conclusions: The Future of the Biocatalysis Pipeline

The case for pursuing marine biocatalysts for incorporation into industrial processes is well established, and the diversity of applications continues to grow. Already there is evidence that the unique geo- and physicochemical properties of the marine ecosystem can provide the evolutionary pressure to select for enzymes with enhanced stress tolerance, activity towards difficult substrates, and improved compatibility with existing industrial processes, overcoming the limitations of early lead catalysts. Therefore, the challenge now is to maximize our ability to extract the key activities that (a) can drive the next generation of industrial processes such as the biopharmaceutical drug synthesis pipeline and (b) open new opportunities based on new biotransformations made possible by the discovery of novel biocatalysts from organisms that to date have proven difficult to capture. This will require integrated and parallel developments in all aspects of the biodiscovery toolkit, from sampling and DNA isolation through to screening and improvement.

From a sequencing perspective, this process has already began with the availability of sequence data encoding enzymes with lower levels of sequence identity than previously seen. This has been driven largely by the isolation and sequencing of metagenomic samples from more diverse environments. This move deeper into “sequence space” is predicted to drive the next phase of sequence-based discovery, taking advantage of the explosion in sequencing projects and improvements in standardized annotation systems. Indeed, some have gone so far as to suggest sequencing of the entire prokaryotic metagenome [23], although the downstream analysis may still prove somewhat of a bottleneck to achieving this.

What is clear is that parallel developments in combinations of the approaches described here will be needed to provide the quantum leap that this broad technology is potentially capable of delivering on. Synergies between bioinformatics, sequencing power, smart screening technologies, and synthetic biology will provide the platform to drive biodiscovery for marine biocatalyst of the future. To achieve this, and maximize its effectiveness, scientists from all disciplines will need to coordinate and cooperate towards common and compatible goals. Expertise in informatics, molecular biology, chemistry, drug design, physics, engineering, systems biology and other disciplines, incorporating both academic and industrial partners will be best placed to address the challenges and provide the future platforms for biocatalytic and natural product discovery.

## Figures and Tables

**Table 1:** Marine microbial biocatalysts discovered based on genome mining and metagenomic approaches.

Marine microbial enzymes	Screening method	Environmental DNA source (G or M)^a^	Reference
Aldehyde reductase	Genome-based	G-*Oceanospirillum* sp. MED92	[67]
Dehalogenase	Genome-based	G-*Psychromonas ingrahamii*	[68]
Lipase (Lip 1)	Genome-based	G-*Pseudoalteromonas haloplanktis*	[69]
Alkane hydroxylase (AlkB)	Function-based	M-Deep sea sediment	[70]
β-Glucosidase (Bgl1A)	Function-based	M-Surface seawater	[71]
β-Lactamase	Function-based	M-Cold seep sediments	[72]
Chitinase	Sequence-based	M-Aquatic habitats	[73]
Chitinase	Function-based	M-Coastal and estuarine waters	[74]
Endo-1,4-Glucanase	Function-based	M-Brown algae	[75]
Esterase (5 different Est)	Function-based	M-Brine:seawater interface	[76]
Esterase (EstA and B)	Function-based	M-Surface seawater	[77]
Esterase (EstAT1 and AT11)	Function-based	M-Seashore sediment	[78]
Esterase/Lipase	Function-based	M-Deep-sea sediment	[79]
Esterase (EstEH1)	Function-based	M-Marine sponge	[80]
Esterase (EstF)	Function-based	M-Sea sediment	[81]
Esterase (EstKT4, T7 and T9)	Function-based	M-Tidal flat sediment	[82]
Esterase (Est6)	Function-based	M-Sea sediment	[83]
Esterase (EstATII)	Function-based	M-Red Sea brine pool	[84]
Esterase (Est97)	Function-based	M-Intertidal zone	[85]
Esterase (EstEP16)	Function-based	M-Deep sea sediment	[86]
Esterase (Est9X)	Function-based	M-Surface seawater	[87]
Fumarase (FumF)	Sequence-based	M-Sea water	[88]
Glycoside hydrolase (GH-57)	Sequence-based	M-Hydrothermal vent	[89]
Glycoside hydrolase (BglMKg)	Function-based	M-Sea water	[90]
Hydrolase (CelM)	Sequence-based	M-Artic ocean	[91]
Laccase (Lac15)	Sequence-based	M-Surface seawater	[92]
Lipase (h1Lip1)	Function-based	M-Sea sediment	[93]
Lipase (LipG)	Function-based	M-Tidal flat sediment	[94]
Lipase (EML1)	Function-based	M-Deep-sea sediment	[95]
Lipase (Lpc53E1)	Function-based	M-Marine sponge	[96]
Lipase (LipA)	Function-based	M-Marine sponge	[97]
Protease	Function-based	M-Sea sediment	[98]
Protease	Function-based	M-Sea sediment	[99]

^a^ G: genomic DNA source; M: metagenomic DNA source.

## Supplementary Files

Supplementary File 1
